# Selenium, zinc, and copper intake and status of vegetarian, vegan, and omnivore children and adolescents: results of the VeChi youth study

**DOI:** 10.1007/s00394-025-03761-3

**Published:** 2025-07-22

**Authors:** Rebecca Simon, Elisa Richter, Kristina Lossow, Morwenna Fischer, Alfred Längler, Andreas Michalsen, Stine Weder, Markus Keller, Anna P. Kipp, Ute Alexy

**Affiliations:** 1 Nutritional Physiology, Institute of Nutritional Sciences, Friedrich Schiller University, Jena, Germany; 2 Faculty of Human Resources, Health and Social Work, University of Applied Sciences (FHM), 33602 Bielefeld, Germany; 3 Department of Paediatrics, Gemeinschaftskrankenhaus, Herdecke, Germany; 4 Faculty of Health, Professorship for Integrativ Paediatrics, Witten Herdecke University, Witten, Germany; 5 Institute of Social Medicine, Epidemiology and Health Economics, Charité- Universitätsmedizin Berlin, 10117 Berlin, Germany; 6 Research Institute for Plant-Based Nutrition, 35444 Gießen/Biebertal, Germany; 7 Department of Nutritional Epidemiology, Institute of Nutritional and Food Science, University of Bonn, 44225 Dortmund, Germany

**Keywords:** Trace element status, Zinc, Selenium, Copper, Children, Adolescents, Vegan diet, Vegetarian diet, Omnivore diet

## Abstract

**Purpose:**

As animal-derived foods are the main source of selenium, zinc, and copper, children and adolescents on vegetarian or vegan dietary patterns are at risk of an inadequate supply.

**Methods:**

Among 342 children and adolescents (6–18 years) with different dietary patterns (86 vegans, 120 vegetarians, 118 omnivores) from the cross-sectional VeChi Youth study serum concentrations of selenium, zinc, and copper and functional biomarkers such as glutathione peroxidase-3 activity (GPX3) and selenoprotein P (SELENOP) for selenium and ceruloplasmin oxidase activity (CPO) for copper were measured. Dietary intake of these trace elements was estimated using a 3-day weighed food record. Group differences were assessed by analysis of covariance, adjusted for age, sex, puberty status, and further covariates.

**Results:**

Trace element intake differed across dietary patterns with lower selenium intake in vegans compared to omnivores (*p* < 0.0001), and lower zinc but higher copper intake in vegans (*p* = 0.0487 and *p* < 0.0001) and vegetarians (*p* = 0.0354 and *p* < 0.0001) than in omnivores. Lower serum selenium as well as SELENOP concentrations were observed in vegans (*p* < 0.0001 and *p* < 0.0001) and vegetarians (*p* < 0.0001 and *p* < 0.0001) in comparison to omnivores, but no difference in GPX3 activity across the dietary groups was observed. Similarly, serum zinc concentrations were lower in vegans (*p* = 0.0122) and vegetarians (*p* = 0.0016) compared to omnivores while serum copper concentrations and CPO did not differ between the dietary patterns.

**Conclusion:**

Vegetarian and vegan dietary patterns are associated with lower intake and serum biomarkers of selenium and zinc and should be monitored in children and adolescents on vegan or vegetarian dietary patterns.

*Trial registration number and date of registration*

DRKS00012835, 11.07.2018.

**Supplementary Information:**

The online version contains supplementary material available at 10.1007/s00394-025-03761-3.

## Introduction

The essential trace elements selenium, zinc, and copper play crucial roles in various physiological processes including growth, development, sexual maturity, and reproduction [1, 2]. Copper enzymes, such as ceruloplasmin oxidase, superoxide dismutase 1, and cytochrome c oxidase, are involved in iron metabolism, redox balance, and mitochondrial ATP production. In addition, many enzymatic reactions important for brain function and the nervous system are catalysed by copper-dependent proteins, including monoamine oxidase or dopamine β-monooxygenase [3]. Zinc, as a component of numerous enzymes and proteins, is closely linked to hormones that promote growth and development, including insulin-like growth factor 1, thyroid hormones, osteocalcin, and sex hormones [4, 5]. Physiological requirements for zinc are the highest during pubertal growth spurt, with an onset at 10 years and peak height velocity at 12 years for girls and a little bit later for boys [6]. Additionally, zinc is vital for the immune system [7], the structure and function of the brain [8, 9], as well as for neurogenesis and neurotransmission [10]. In the form of zinc finger proteins, zinc contributes to genetic stability and acts as a regulator of gene expression [5]. Selenium, incorporated as selenocysteine, is essential for the expression and activity of selenoproteins. Glutathione peroxidases (GPX) and thioredoxin reductases are selenoproteins important for maintaining redox homeostasis, while iodothyronine deiodinases catalyse thyroid hormone synthesis [11]. An adequate selenium status during the prenatal phase and in childhood has been associated with children’s cognitive function later in life [12]. The status of all three trace elements is primarily characterized by analyzing serum concentrations of zinc, copper, and selenium, while for the latter two also functional biomarkers (GPX3 activity and selenoprotein P in case of selenium and ceruloplasmin oxidase activity in case of copper) are suggested to be analysed in parallel [13]. As meat and other animal-derived foods are the primary sources of those trace elements, individuals following plant-based diets are at increased risk for an insufficient intake and status.

The interest in plant-based diets continues to grow not only among adults, but also among children and adolescents. In the “Eating study as a KiGGS Module” (EsKiMo II), 1.5% of the 6 to 11-year-olds and 5.1% of the 12 to 17-year-olds adhered mainly to a vegetarian diet [14]. Although vegetarian (excluding meat and fish) and vegan (excluding all animal-derived foods) diets are supposed to protect from a number of chronic diseases such as cardiovascular disease, obesity, diabetes, and some tumors [15], the exclusion of certain foods from the diet may lead to inadequate nutrient intake, especially in more restrictive forms such as vegan diets [16].

In the vulnerable group of children and adolescents, caution is required when it comes to ensuring that all nutrients are adequately supplied through a vegan diet, as energy and nutrient intake per kg of body weight is generally higher than in adults to ensure normal growth and development [17]. As there is limited evidence for health effects of vegan diets in paediatric populations, the German Nutrition Society (DGE) in their position paper from 2024 draws attention to the potential risks and thus does not make a clear recommendation either in favor of or against a vegan diet in vulnerable groups such as children and adolescents. Specifically, the DGE classifies the vitamins D, B_2_, B_12_, as well as calcium, and the trace elements iron, iodine, zinc, and selenium as critical nutrients in a vegan diet [18]. Hence, it is important to investigate the supply and status of these nutrients in children and adolescents following a vegan or vegetarian diet. To our knowledge, this is the first study to investigate in parallel the zinc, selenium, and copper intake and status of children and adolescents across a wide age range (6–18 years), comparing not only vegetarians and omnivores but also vegans.

## Methods

### VeChi youth study

The cross-sectional VeChi Youth Study examined anthropometrics, diet, lifestyle, and nutritional status of German vegetarian, vegan, and omnivore children and adolescents (*n* = 401) between October 2017 and January 2019 [19]. The study was conducted according to the guidelines of the Declaration of Helsinki and approved by the ethics committee of the University of Witten-Herdecke (139/2017). The study has been registered at the German Clinical Trials Register (DRKS00012835). The implementation of the VeChi Youth Study was commissioned within the scope of the 14th DGE Health Nutrition Report, published by the DGE and financed by the German Federal Ministry of Food and Agriculture (BMEL).

### Participants

Healthy vegetarian, vegan or omnivore children and adolescents (aged 6–18 years) living in Germany were recruited mainly via the study website (www.vechi-youth-studie.de), social media groups on plant-based diets, flyers, magazines, journals, and vegetarian or vegan societies’ websites [19]. Exclusion criteria were (a) diagnosed diseases that could affect the studied variables (e.g., enteropathy, pancreatic diseases, metabolic disorders like phenylketonuria or fructose malabsorption), and (b) special diets other than vegan or vegetarian diet, e.g., predominantly (≥ 70%) raw food diets, as food selection is further restrained (e.g. no legumes, no potatoes) [19]. The adherence to one of the three dietary patterns was assessed by an online questionnaire during recruitment (“do you/does your child eat a vegetarian diet (no meat, sausage, fish, but dairy and/or eggs), a vegan diet (nor meat, sausage, fish, dairy, and eggs), or an omnivore diet (including meat and/or fish)?”). The classification was additionally cross validated by an online food frequency questionnaire [19].

### Study procedure

All participants were invited to one of three study centers (Department of Integrative Paediatric and Adolescent Medicine, Gemeinschaftskrankenhaus, Herdecke, in the West of Germany, Department of Internal and Integrative Medicine, Immanuel Krankenhaus Berlin, in the East of Germany or Filderklinik, Filderstadt-Bonlanden, in the South of Germany). During the study center visit, anthropometric measurements were performed, venous blood samples (< 20 ml), and spot urine samples were collected. Additionally, an online questionnaire on sociodemographic lifestyle variables was completed and a 3-day weighed food record was conducted at home. Study participants received an incentive of 50 € each and were provided with the results of the dietary records and blood analyses [19].

### Laboratory measurements

Blood samples were centrifuged (10 min, 2500 x g, 20 °C) for separation of serum and stored at − 80 °C until analysis.

### Dietary assessment

At study center visit, families got written information on dietary recording. The participating families choose the day of the beginning of dietary recording within a given period. All foods, beverages and leftovers were weighed and recorded over 3 days using electronic kitchen scales by the parents and/or the older participants themselves. If weighing was not possible (e.g., in case of eating out), participants were asked for semi-quantitative recording using household measures (e.g. spoons, cups). Packages of commercial food products should be collected, too, either as a photo or the packaging was sent by mail. After recording, missing data or uncertainties were clarified by the study staff requesting the information from the participants via e-mail. Energy and nutrient intake (i.e., zinc and copper) was calculated as individual mean of three recording days using the in-house food composition database LEBTAB [20]. The basis of LEBTAB is the German standard food composition database (Bundeslebensmittelschlüssel, BLS). In addition, LEBTAB is continuously updated by adding all commercial food products or nutrient supplements recorded by study participants, e.g. ready-to-eat meals, desserts and snacks, including milk, meat or fish alternatives. Hereto, the energy and nutrient contents were estimated by recipe simulation using labelled ingredients and nutrient contents including fortification. As LEBTAB does not contain data on selenium, the recorded foods were linked to the selenium food concentrations provided by the European Food Safety Authority (EFSA) food composition database [21, 22], as described in [23]. Dietary supplements reported in the dietary records were not considered for this analysis.

### Assessment of covariables

Body weight (Seca 799, graduation 100 g, up to 150 kg body weight) and height (stadiometer Seca 222, graduation 1 mm) were measured in underwear, without shoes by trained staff. The Body Mass Index (BMI) was calculated from body weight and height. The standard deviation score of BMI (SDS-BMI) was calculated according to the LMS method using the German reference values [24].

Socio-demographic variables (i.e., place of residence in large (> 20.000 inhabitants) or small towns/villages, household income per months, parental education, and profession), and lifestyle variables, (i.e., smoking in the household, the overall use of nutrient supplements (“does your child/do you take nutrient supplements?”), if yes, “do these supplements contain selenium/zinc?”), and the duration of the adherence to the dietary pattern were assessed using an online questionnaire. Data on physical activity was collected with the validated Adolescent Physical Activity Recall Questionnaire [25].

For the categorization of socioeconomic status (SES) an established index [26] was calculated, combining income, education, and profession (1–7 points, each). A score of 3–8 points indicated a low SES, 9–14 points a middle SES, and 15–21 points a high SES. MET-minutes per week were calculated from information provided about organised and unorganised activities and databases on the metabolic intensity of activities [27, 28].

Puberty stages according to Tanner were assessed by a questionnaire including schematic drawings [19]. Subjects were classified as pre-pubertal (Tanner = 1) and pubertal (at least one criterion of Tanner stage 2–5). Three of five missing values were classified as pubertal (two girls aged 17 and 19 years) or pre-pubertal (one boy aged 6 years) by researchers.

### Analysis of trace elements in serum samples

Trace element concentrations in serum samples were determined by total X-ray fluorescence (TXRF) using a benchtop TXRF spectrometer with a molybdenum X-ray tube (S4 T-Star™, Bruker Nano GmbH, Berlin, Germany) [29]. A gallium solution (1 mg/mL, Thermo Fisher Scientific, Kandel, Germany) was added to all samples as an internal standard. 10 µL of each solution was placed onto quartz carriers and dried at 40 °C on a heating plate before measuring for 750 s. In total, only 22 µl serum is needed for duplicate analyses. For quality control, reference serum was measured in the same manner during each measurement cycle (ClinChek^®^ serum control level I Ref. 8880 Lot 544, RECIPE Chemicals + Instruments, Munich, Germany). All values were in the expected range.

### Enzymatic activity of GPX3 and CPO

GPX3 activity measurement in serum was conducted via a NADPH-consuming glutathione reductase coupled assay as previously described [30]. Briefly, hydrogen peroxide (Merck KGaA, Darmstadt, Germany) is reduced by GPX enzymes, leading to the concomitant oxidation of glutathione (Merck/Sigma-Aldrich, Darmstadt, Germany). The oxidised glutathione is then converted back to its reduced form via a coupled reaction with glutathione reductase (Merck/ Sigma-Aldrich) and NADPH (Carl Roth, Karlsruhe, Germany). The reduction of NADPH is proportional to GPX activity. Absorbance was measured at 340 nm using a microplate reader (Synergy H1, Agilent Technologies/BioTek, Santa Clara, CA, USA) with samples diluted 1:5 and analysed in triplicate.

Ceruloplasmin oxidase activity (CPO) in serum was determined as described before [31]. O-dianisidine dichloride (VWR/Thermo Scientific Chemicals, Radnor, PA, USA) used as a substrate is oxidised by ceruloplasmin to a chromophore in the presence of oxygen. After the addition of 9 M sulphuric acid (Merck KGaA, Darmstadt, Germany) the absorbance of the purplish-red solution was measured spectrophotometrically at 550 nm at two different time points: 10 min in single and 60 min in triplicate using a microplate reader (Synergy H1, Agilent Technologies/BioTek). Samples were diluted 1:3.

The enzyme activities are expressed as U/L.

### SELENOP ELISA

A validated, commercial sandwich ELISA (selenOtest™, SelenOmed GmbH, Berlin, Germany) was used to determine Selenoprotein P (SELENOP) levels. Sample preparation and assay procedure were carried out as specified by the manufacturer.

### Statistical analysis

The statistical analysis was performed using SAS^®^ procedures (version 9.4). The significance level was set to *p* < 0.05. All data were checked for plausibility and outliers. To describe sample characteristics, medians and quartiles, or frequencies and percentages were calculated, stratified for the three dietary patterns.

**Fig. 1:**
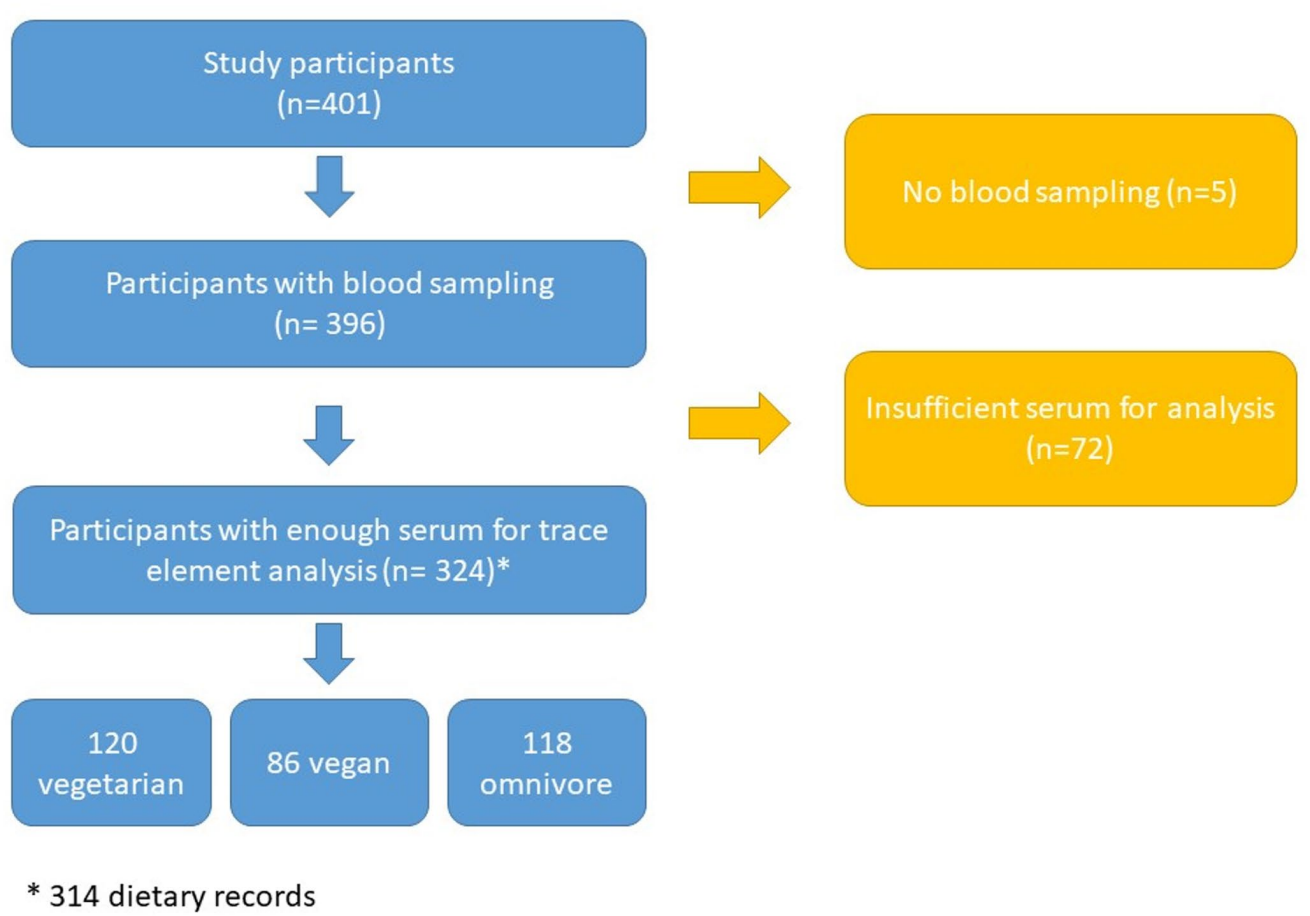
Flow chart of the study participants

To compare means in the main outcomes (i.e., biomarkers and intakes of selenium, copper, zinc) across dietary patterns, we used analysis of covariance with the vegetarians or vegans compared with omnivores (reference group). All outcome variables were log-transformed to fulfill model assumptions. Outliers, i.e., values > 1.5 times interquartile range above the third quartile or < 1.5 times the interquartile range below the first quartile of biomarker and intake data were winsorized. The selection of covariates based on directed acyclic graphs (DAG) [32–34]. Including age, sex, BMI-SDS, intake of dietary supplements, intake of selenium supplement, duration of adherence to dietary pattern, physical activity level, socio-economic status, total energy intake, and smoking status, DAG identifies three covariables (i.e., age, sex, socio-economic status) as a minimum set of potential confounders to control for (Fig. 1). Multiple imputation was used to deal with missing values of covariates assuming of missing at random. *p*-values were adjusted for multiple testing using the False Discovery Rate method (Proc Multitest in SAS).

Due to the physiological changes in the metabolism of the trace elements, the analyses of the biomarkers were carried out both in the overall collective and stratified according to puberty status. A sensitivity analysis was carried out for zinc, in which additional adjustments were made for the intake of dietary fiber (g/day) (as a proxy for the intake of phytate), but this did not lead to substantial changes in the results (data not shown). To compare biomarker and intake data of trace elements, spearman rank correlation coefficients were calculated. In addition, participants were grouped into quantiles for age and sex adjusted residuals of biomarker and intake data, respectively. The degree of agreement was evaluated using the weighed kappa coefficient (κ). Furthermore, dietary intake data were calculated as percentage of age- and sex-specific German reference values [35].

## Results

### Study sample

Of the 396 blood samples, 324 serum samples were still available for the analysis of all biomarkers for selenium, zinc, and copper (Fig. 1). Hence, the final sample consists of 120 vegetarians, 86 vegans, 118 omnivores children and adolescents (Table 1). Median age was 13 years across all dietary groups, more than half of the participants were girls (*n* = 182, 56.2%), but the percentage was significantly higher in the vegan and vegetarian groups in comparison to the group of omnivores. There was no significant difference in body weight, height, SDS-BMI, and total energy intake between diet groups, but intakes of protein, fat, carbohydrates, and dietary fiber differed. While protein and fat intake both were higher in omnivores, overall carbohydrates and fiber intake were lower in comparison to vegans and vegetarians. In all dietary groups, more than 80% of participants lived in large towns, and reported a high SES, which appears to be the lowest in the vegan group. The prevalence of smoking in the household was low. The unadjusted biomarker and intake data are provided in Table S1.

The overall intake of supplements, as indicated by the online questionnaire, differed significantly between diet groups, with nearly all vegans reporting supplement intake (Table 1). Accordingly, the prevalence of selenium (7.1%) and zinc supplementation (16.7%) was highest in vegan participants (*p* < 0.05). None of the participants reported copper supplementation.

**Table 1:** Sample characteristics of children and adolescents (6–18 years) from the VeChi youth study (*n* = 324)

	Vegan (*n* = 86)	Vegetarian (*n* = 120)	Omnivore (*n* = 118)	*p*-value ^a^
*Characteristics*				
Age, y	13.7 (9.6; 17.2)	13.4 (9.7; 16.4)	13.3 (10.0; 16.3)	0.7180
Sex (girls)	55 (64.0)	73 (60.8)	54 (45.8)	**0.0152**
Pre-pubertal	41 (35.0)	32 (27.4)	44 (37.6)	0.9032
Pubertal	77 (37.6)	54 (26.3)	74 (36.1)	
*Anthropometrics*				
Body height, cm	157 (136; 171)	160 (138; 171)	162 (141; 174)	0.3482
Body weight, kg	45 (28; 57)	47 (31; 60)	45 (32; 61)	0.4304
SDS-BMI	-0.62 (-1.13; 0.14)	-0.22 (-0.83; 0.29)	-0.24 (-1.0; 0.35)	0.0589
*Dietary intake* ^b^				
Total energy, kcal	1657 (1364; 1950)	1708 (1330; 1991)	1737 (1431; 2143)	0.1741
Protein, %E	11.9 (10.7; 13.8)	11.4 (10.2; 12.7)	13.2 (11.8; 15.3)	**< 0.0001**
Protein, kg BW	1.2 (0.8; 1.7)	1.1 (0.9; 1.5)	1.3 (1.1; 1.6)	**0.0044**
Carbohydrates, %E	57.2 (50.7; 62.2)	54.6 (50.2; 59.2)	48.7 (44.7; 54.8)	**< 0.0001**
Fat, %E	29.1 (24.6; 37.0)	32.4 (28.3; 37.5)	36.6 (30.4; 41.2)	**< 0.0001**
Fiber, g/1000 kcal	22.8 (17.8; 26.0)	14.3 (11.2; 17.9)	11.9 (9.9; 14.0)	**< 0.0001**
Duration adherence to dietary pattern, y	2.9 (1.7; 5.1)	4.1 (2.2; 6.7)	-	**0.0180**
*Sociodemographic and lifestyle characteristics* ^c^				
Residence large town ^d^	69 (82.1)	96 (87.3)	93 (83.0)	0.5583
High SES	50 (60.2)	83 (72.2)	92 (81.4)	**0.0047**
Smoking in the household	5 (6.0)	7 (6.1)	2 (1.8)	0.2172
Physical activity, MET minutes	1022 (630; 1685)	1090 (662; 1640)	1137 (741; 1718)	0.9501
*Supplement use*				
All supplements	81 (96.4)	55 (48.3)	19 (16.8)	**< 0.0001**
Trace element specific supplements				
Selenium	6 (7.0)	4 (3.3)	5 (4.2)	**0.0353**
Zinc	14 (16.7)	5 (4.4)	4 (3.5)	**< 0.0001**

Values are median (Q1; Q3) or numbers (%), %E percent of total energy intake, SES socio-economic status.

^a^ Kruskal–Wallis tests or χ2 tests were used to test for group differences.

^b^ Calculated as individual means of three record days. No dietary record available for 10 participants (5 vegetarians, 3 vegans, 2 omnivores).

^c^ missing data: residence: *n* = 18, Socioeconomic status: *n* = 13 smoking in the household: *n* = 12, supplement use: *n* = 13, selenium supplement use: *n* = 2, physical activity: *n* = 6.

^d^ >20,000 inhabitants.

**Table 2:** Adjusted means, standard errors (SE), and adjusted mean differences (%) of trace element (selenium, copper, zinc) biomarker and dietary intake data between vegetarian, vegan, and omnivore children and adolescents (6–18 years) from the Vechi youth study for the total sample (*n* = 324) and stratified by puberty (pre-pubertal *n* = 117/pubertal *n* = 205)

	Vegan	Vegetarian	Omnivore	Vegan^b^		Vegetarian^b^	
	Adjusted ^a^ mean (SE)	Adjusted ^a^ mean (SE)	Adjusted ^a^ mean (SE)	Δ (95% CI)	p-value	Δ (95% CI)	p-value
**Biomarker**							
*Selenium* (µg/L)							
Total Sample	64.1 (1.5)	62.9 (1.7)	70.8 (1.5)	-0.11 (-0.17; -0.06)	**0.0004**	-0.15 (-0.21; -0.09)	**0.0004**
Pre-pubertal	58.1 (2.4)	59.3 (2.6)	69.0 (2.2)	-0.17 (-0.26; -0.09)	**0.0004**	-0.17 (-0.26; -0.08)	**0.001**
Pubertal	67.3 (2.0)	65.2 (2.4)	71.8 (2.0)	-0.08 (-0.15; -0.02)	**0.0277**	-0.14 (-0.21; -0.07)	**0.0007**
*SELENOP (mg/L)*							
Total Sample	2.70 (0.07)	2.31 (0.08)	3.24 (0.07)	-0.20 (-0.27; -0.13)	**0.0004**	-0.39 (-0.46; -0.30)	**0.0004**
Pre-pubertal	2.75 (0.12)	2.44 (0.13)	3.27 (0.11)	-0.18 (-0.30; -0.07)	**0.0056**	-0.34 (-0.46; -0.21)	**0.0004**
Pubertal	2.71 (0.09)	2.25 (0.10)	3.24 (0.08)	-0.20 (-0.29; -0.11)	**0.0004**	-0.42 (-0.51; -0.32)	**0.0004**
*GPX3 (U/L)*							
Total Sample	166 (3)	165 (4)	178 (3)	-0.07 (-0.13; -0.01)	**0.0330**	-0.09 (-0.15; -0.02)	**0.0185**
Pre-pubertal	163 (6)	169 (7)	172 (6)	-0.06 (-0.16; 0.04)	0.3015	-0.01 (-0.12; 0.09)	0.1153
Pubertal	171 (4)	162 (5)	182 (4)	-0.07 (-0.15; 0.01)	0.1146	-0.13 (-0.21; -0.04)	**0.0074**
*Copper (µg/L)*							
Total Sample	942 (26)	950 (31)	943 (26)	-0.00 (-0.06; 0.06)	0.9340	0.00 (-0.07; 0.07)	0.9362
Pre-pubertal	936 (37)	1026 (42)	1043 (36)	-0.11 (-0.20; -0.01)	**0.0500**	0.01 (-0.09; 0.11)	0.9340
Pubertal	927 (35)	874 (42)	884 (34)	0.03 (-0.05; 0.11)	0.5782	-0.02 (-0.10; 0.07)	0.8074
*CPO (U/L)*							
Total Sample	110 (4)	107 (5)	109 (4)	0.02 (-0.07; 0.10)	0.8074	-0.02 (-0.11; 0.08)	0.8074
Pre-pubertal	112 (5)	119 (6)	125 (5)	-0.13 (-0.26; 0.01)	0.1021	-0.02 (-0.17; 0.12)	0.8074
Pubertal	106 (5)	96 (6)	100 (5)	0.07 (-0.05; 0.18)	0.3234	-0.03 (-0.15; 0.10)	0.8054
*Zinc(µg/L)*							
Total Sample	822 (15)	795 (18)	873 (15)	-0.06 (-0.10; -0.01)	**0.0270**	-0.08 (-0.12; -0.03)	**0.0048**
Pre-pubertal	797 (25)	776 (28)	849 (24)	-0.07 (-0.14; 0.01)	0.1139	-0.08 (-0.16; 0.01)	0.1139
Pubertal	836 (20)	811 (23)	885 (19)	-0.05 (-0.10; 0.01)	0.1172	-0.08 (-0.13; -0.02)	**0.0270**
**Intake**							
Selenium (µg/d)	31.0 (2.7)	38.1 (3.2)	44.6 (2.7)	-0.25 (-0.34; -0.16)	**0.0004**	-0.07 (-0.17; 0.03)	0.2593
Copper (mg/d)	1.6 (0.2)	2.2 (0.2)	1.7 (0.2)	0.17 (0.10; 0.23)	**0.0004**	0.49 (0.41; 0.56)	**0.0004**
Zinc (mg/d)	8.1 (0.3)	9.2 (0.3)	9.1 (0.3)	-0.06 (-0.11; -0.00)	0.0852	0.07 (0.00; 0.13)	0.0646

^a^ Means (SE) adjusted for age and sex, GPX3 glutathione peroxidase-3 activity, SELENOP selenoprotein P, CPO ceruloplasmin oxidase activity

^b^ Outlier of outcome variables were winsorized. Outcome variables have been log-transformed to account for skewness of raw data and to fulfill model requirements. Multiple imputation was used to account for missing covariables data, model adjusted for age, sex, puberty (pre-pubertal/pubertal) and socioeconomic status, models for intake were also adjusted for total energy intake per day. *p*-values were adjusted for multiple testing using the False Discovery Rate method (Proc Multitest in SAS).

### Biomarker

Both the serum selenium and SELENOP concentration differed significantly between vegan and omnivore as well as vegetarian and omnivore children and adolescents. Omnivore participants showed the highest levels for both parameters, vegetarian participants the lowest, which might be related to the higher supplement intake in the vegan group. GPX3 activity was highest in omnivores and lowest in vegetarians, and group differences were significant between vegetarian and omnivore participants and between vegans and omnivores. Additionally, pre-pubertal subjects had lower selenium concentrations than pubertal subjects within each dietary group, whereas SELENOP levels were higher in pre-pubertal individuals (Table 2).

Serum zinc concentrations were highest in omnivores and lowest in vegetarians. Both vegetarians and vegans differed significantly from omnivores. In each dietary group, pre-pubertal individuals had lower concentrations than the older participants (Table 2). Interestingly, the effects of dietary patterns on serum zinc levels were not modulated by additionally adjusting for fiber intake as a proxy for phytate intake in the sensitivity analysis.

No significant group differences were found for copper biomarkers. Pre-pubertal vegetarians and omnivores had higher copper concentrations and CPO activities than the pubertal individuals (Table 2).

### Intake

The estimated dietary intake of selenium was highest in omnivore and lowest in vegan participants. Group differences were significant between vegans and omnivores, but not between vegetarian and omnivore participants. The zinc intake was highest in vegetarians and lowest in vegans, but group differences were not significant. Copper intake was highest in vegetarian and lowest in vegan participants and differed significantly in comparison with omnivore participants.

**Fig. 2:**
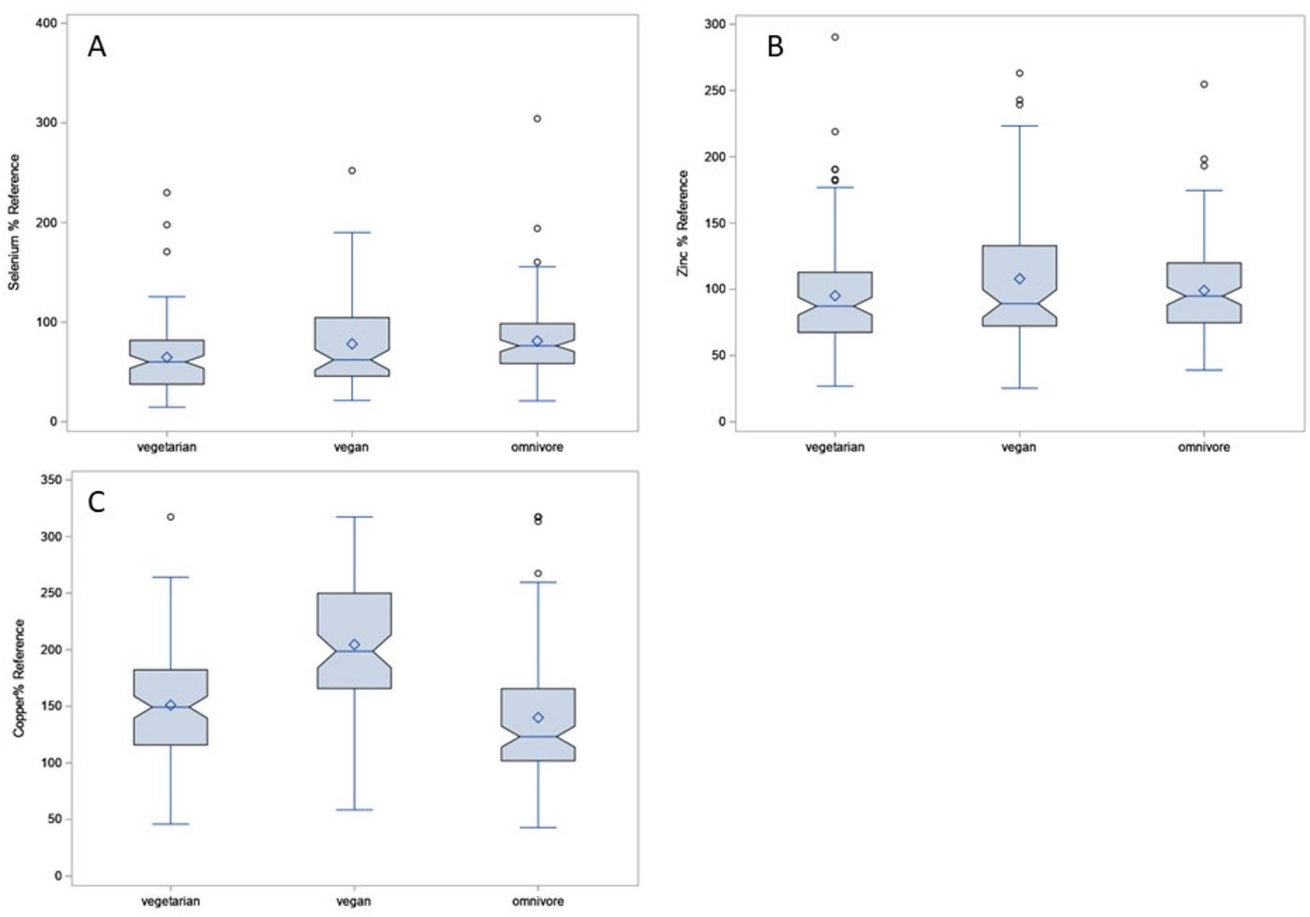
Selenium (A), zinc (B), and copper (C) intake as % of the German age- and sex-specific reference value in vegetarian, vegan, and omnivore participants of the VeChi Youth Study (6–18 years of age, 2017–2019) [35]

Compared to the German reference values, median selenium intake was below the reference values in all groups (vegetarian: median (Q1; Q3) 60% (38%; 82%); vegan: 62% (46%; 105%); omnivore: 76% (58%: 99%). The same was the case for median zinc intake (vegetarian 87% (68%; 113%); vegan: 89% (72%; 133%), omnivore: 95% (75%; 120%). In case of copper, most participants exceeded reference values (vegetarian: 149% (116%; 182%); vegan: 199% (166%; 250%); omnivore 123% (102%; 166%) (Fig. 2).

### Comparison between biomarkers and intake data

Except for selenium intake, there was no significant correlation between dietary intake data and serum biomarkers of the same nutrient. Partial correlation coefficients were low (< 0.3) for selenium intake and serum selenium or SELENOP concentrations. Also ranking the participants according to their estimated intake and biomarker level was slight (κ 0.00-0.20) for all biomarkers, except for SELENOP (κ = 0.24), indicating a fair agreement (Table 3).

**Table 3:** Agreement between dietary intake data and serum biomarkers of trace elements (each calculated as age- and sex-adjusted residuals) in vegetarian, vegan, and omnivore children and adolescents (6–18 years) from the Vechi youth study with both dietary records and biomarker data (*n* = 314)

	Partial correlation coefficient	*p*-value	κ ^a^	95% CI
Selenium intake				
Concentration	0.19	**0.0007**	0.19	0.09; 0.30
SELENOP	0.29	**< 0.0001**	0.24	0.13; 0.35
GPX3	0.11	0.0587	0.13	0.02; 0.24
Copper intake				
Concentration	-0.10	0.0773	-0.09	-0.20; 0.02
CPO	-0.10	0.0622	-0.12	-0.22; -0.01
Zinc intake				
Concentration	0.03	0.6514	-0.00	-0.12; 0.11

GPX3 glutathione peroxidase-3 activity, SELENOP selenoprotein P, CPO ceruloplasmin oxidase activity.

^a^ Strength of the agreement: < 0 poor, 0-0.20 slight, 0.21–0.40 fair, 0.41–0.60 moderate, 0.61–0.80 substantial, 0.81-1.00 perfect [36].

## Discussion

Exclusion of certain foods results in different nutrient profiles, leading, besides others, to variations in trace element intake and status. Herein, we could show that dietary patterns with a different degree of consumption of animal-derived foods (vegan, vegetarian, and omnivore) of children and adolescents clearly affected the intake of selenium, zinc, and copper. While a higher copper intake had no effect on serum copper biomarkers, a lower intake of selenium and zinc resulted in the reduction of the respective serum biomarkers with putative functional consequences.

Previous studies in adults have shown that individuals following a vegetarian or vegan diet tend to have a lower selenium intake [16, 37–39]. In Europe, the soil is largely selenium-poor, resulting in low selenium content of regionally grown plant-based foods. In contrast, animal feed is fortified with selenium in the European Union, making animal-derived foods, particularly meat, eggs, and fish, the primary sources of dietary selenium [40, 41]. Consequently, omnivores generally consume higher amounts of selenium than vegans or vegetarians [16, 37–39]. In line with results for adults, vegan, and vegetarian children and adolescents of our study had a reduced selenium intake, but only vegans consumed significantly less selenium per day compared to omnivores. A lower intake in vegans was also observed in a study of 16-20-year-olds [42]. In contrast in the VeChi Diet study (*n* = 430, 1–3 years of age, 2016–2018) vegetarians had a lower selenium intake than vegans. This was explained by a higher consumption of Brazil nuts (one of the most selenium-rich plant-based foods) and legumes in vegans, which accounted for 19% and 17% of total selenium intake, respectively. The younger children reached the harmonized adequate requirement of 17 µg/day on average in all dietary patterns [23]. However, in the present study, selenium intakes, regardless of dietary pattern, were mostly below the European recommended daily intakes of 35 µg for children aged 7–10 years, 55 µg for adolescents aged 11–14 years, and 70 µg for adolescents aged 15–18 years [43]. This raises concerns about a general suboptimal selenium supply. However, the selenium intake is still only an estimate, as the EFSA database used for intake calculation is limited to the selenium content of foods produced in Europe and does not reflect deviating contents of imported foods [22]. Furthermore, the higher selenium supplement use among vegans in the VeChi-Youth-Study, as indicated by the online questionnaire, but not considered for selenium intake, is notable and might confound the results of dietary assessment. Despite these limitations, there was a strong correlation between the estimated selenium intake and serum selenium concentrations and especially with the functional biomarker SELENOP (κ > 0.2). Therefore, the estimated selenium intake appears to be a useful indicator for assessing the individual selenium status.

Due to the limitations in the estimation of dietary selenium intake, functional biomarkers should be considered to assess the selenium status. Unlike the total serum selenium concentration, which includes all forms of selenium (free or bound to selenoproteins or other proteins), serum biomarkers such as SELENOP concentration and GPX3 activity provide an information about the functionally available selenium for selenoprotein synthesis [13]. Serum SELENOP concentration is considered to be a sensitive biomarker, as in adults a maximum is only reached at a selenium concentration of 100–120 µg/l, while serum GPX activity already reaches a maximum at a concentration of 70–90 µg/l [44–48]. As the concentrations of SELENOP decreases more rapidly than GPX3 when the supply is reduced, it is also more sensitive to a suboptimal selenium status [49]. Our findings indicate that vegan and vegetarian children and adolescents exhibited significantly lower values across all three selenium biomarkers compared to omnivores. Vegetarians have the lowest values which might be due to the higher frequency of selenium supplementation among vegans. Also in other cohorts, young female vegetarians showed significantly lower selenium concentrations than non-vegetarians [50]. Similarly, in children of about 5 years of age selenium intake decreased with vegan or vegetarian dietary habits. Also in this age group, vegetarians had the lowest selenium concentrations, lower than vegans and omnivores, but this was not statistically significant [51]. However, it is unclear whether supplements were considered in this analysis. In our study, vegans reported a more frequent supplementation of selenium than other groups but only intake from dietary sources was included in the estimation of the selenium intake due to the high number of missing values. A previous study highlighted that among vegan adolescents, selenium supplements significantly contributed to the selenium status accounting for approximately 50% of total intake [52].

In the present study, the lowest daily zinc intake from food was observed in vegans while vegetarians and omnivores were in a comparable range. Especially for adolescents, the values were below the EFSA recommendations for zinc intake (7.4 mg/day for 7–10-year-olds, 10.7 mg/day for 11–14-year-olds and 11.8 mg/day for 15–17-year-old males and 14.2 mg/day for females) [53]. Previous studies have found lower zinc intakes among vegetarian children and adolescents than non-vegetarians, although the differences were not always statistically significant [42, 54–57]. The main sources of zinc for German children and adolescents were bread, meat, and milk, which together accounted for approximately 40% of intake [58]. Among Swedish vegan adolescents, dietary supplements represent a significant source of zinc, contributing to approximately one fourth of total zinc intake [52]. Adolescents adhering to a vegan diet exhibit lower zinc intake than their omnivore counterparts, when only food sources were considered [42]. Differences of the daily zinc intake between an omnivore diet and a vegetarian or vegan diet were 1 mg and 2 mg, respectively [59].

However, not only the absolute amount of zinc intake is of relevance but rather its bioavailability which depends on various dietary components. Phytate is found in high amounts in plant-derived foods such as cereals, legumes, and grains. Data on phytate intake in children and adolescents were not available for this study. However, as plant foods are also the main source of dietary fiber, it can be assumed that vegans also consumed the highest amount of phytate, as significant differences in dietary fiber intake were observed between the different dietary patterns, with the highest amounts in vegans, followed by vegetarians [60]. Phytate can reduce intestinal zinc absorption by forming insoluble complexes, thereby limiting its bioavailability [61]. Hence, the German and European zinc reference values for adults, but not for children, are stratified by phytate intake levels [53, 62]. In younger children up to 4 years of age, no effect of phytate intake on zinc absorption could be determined [63]. Based on this study, the reference values for children and adolescents were not stratified by phytate intake but instead the average absorption rate of zinc from an omnivore middle-European diet of 31% [62] was used for calculation. However, the absorption rate is estimated to be only 23% from vegetarian/vegan diets with high phytate intake [64, 65]. A meta-analysis reported that children and adolescents of all three dietary patterns met recommended zinc intakes, but when adjusting for the lower bioavailability from phytate-rich diets, some studies report that there was not sufficient zinc intake from food alone [59].

Due to these differences in bioavailability, it is important to analyse biomarkers to assess the zinc status, i.e., serum zinc concentration [66, 67]. Most studies in children and adolescents did not find significant differences in serum zinc concentration between dietary patterns [50, 51, 54], except of a Finish study with a small sample of younger children which found significantly lower zinc concentrations in vegetarians but not vegans in comparison to omnivores [17]. We observed significantly lower zinc concentrations in vegetarians as well as vegans than in omnivores. The serum zinc concentrations of our participants (795–873 µg/L) fell within the paediatric reference interval of 640–1240 µg/L [68]. The lack of a significant correlation of zinc intake and serum zinc concentration in our study can be explained by differences in bioavailability and physiological zinc homeostasis, as serum zinc concentration is tightly regulated by absorption and excretion [67]. Even on a restrictive diet with zinc intakes as low as 3–4 mg/day, plasma concentrations remain stable in adults, indicating that concentrations not necessarily decrease with lower zinc intake [69]. Other factors such as time of day, fasting state, exercise, infection and inflammation, or stress can affect zinc levels, resulting in a fluctuation of 20% within 24 h [70].

In contrast to the other two trace elements, plant-derived foods, mainly cereals and their products, fruits, and vegetables are important contributors to copper intake [56, 71]. In agreement with the literature, our analysis indicates that copper intake was significantly higher among vegetarians compared to omnivores, findings that have been observed across adults, children, and adolescents, although not always significant [37, 56, 72]. In contrast, in adults, others have reported the highest intakes in vegans [30], whereas we showed significantly lower intakes in children and adolescents on a vegan than omnivore diet. Notably, all participants irrespective of the dietary pattern exceeded the adequate intake of 1 mg/day for 3–9-year-olds and 1.3 or 1.1 mg/day for 10-17-year-old males and females [73], but remaining below the age-dependent upper limits of 2–4 mg/day, as established by the Scientific Committee on Food (SCF) [74]. Despite variations in copper intake among dietary groups, there were no significant differences in serum copper concentration or CPO across vegan, vegetarian, and omnivore diets in children and adolescents. Ceruloplasmin is the major copper-carrying protein in the blood, binding approximately 95% of circulating copper [75]. In adults, lower serum concentrations and ceruloplasmin activity were associated with a vegan/vegetarian diet though not significantly different [30]. Copper homeostasis is tightly regulated, preventing excessive copper accumulation and its associated oxidative potential, which could otherwise lead to cellular damage through free radical formation [76]. Consequently, our study did not find significant correlations between dietary copper intake and serum copper concentrations or ceruloplasmin activity, as also described in previous studies [77]. In the study by Hunt et al., serum copper and ceruloplasmin concentrations were unaffected by diet [78], which fits to the concept that intestinal uptake is not mainly affecting copper homeostasis but rather its excretion into the bile. Aside from that, other factors influence serum copper and ceruloplasmin concentrations. In children, the highest serum copper concentrations are observed in younger ages, with levels decreasing during growth until puberty [79]. During puberty, copper concentrations decline in males but remain stable in females [80, 81]. Gender differences in serum copper concentrations are particularly evident during the reproductive years, where higher concentrations are observed in women between 20 and 30 years of age [80]. These elevated copper levels in women are probably due to estrogen and therefore also the use of hormonal contraceptives significantly increases serum copper levels as well as ceruloplasmin [82–84]. Recommended reference intervals range from 889 to 2891 µg/L, with the lowest values in infants and the highest in women 15–50 years of age using hormonal contraceptives [79].

The study has several strengths which include particularly the large sample size covering a wide age range, the inclusion of three different dietary patterns, and the analysis of not only trace element intake but also serum concentrations and biomarkers, which allows for a more comprehensive assessment of trace element status. However, there are also some limitations. First, the cross-sectional study design does not allow for causal conclusions. Secondly, these results may not be generalizable or representative due to a potential selection bias. In the study, mainly families with high SES participated, especially in the omnivore group. It is well known that SES has an influence on food choice, diet quality, and children’s health [85, 86]. Furthermore, even if the study used a well-established and detailed dietary assessment method, the self-reported instrument may have caused a diet measurement error [19].

In conclusion, dietary patterns in children and adolescents significantly influence their trace element profiles. While modulation of copper intake is well within the adequate range, reduced intake of selenium and zinc might be critical in children and adolescents on a vegetarian or vegan diet and could be monitored if necessary. This highlights the need for carefully planning long-term vegetarian and vegan diets, including the use of supplements to ensure adequate trace element status in children and adolescents. Longitudinal studies with children and adolescents on a vegan or vegetarian diet would be desirable to investigate possible associations between nutrient intake and health.

## Electronic supplementary material

Below is the link to the electronic supplementary material.

Supplementary Material 1

## Data Availability

Data of the VeChi Youth Study are available on request to alexy@uni-bonn.de.

## Declarations

(139/2017).
