# Correction: Selenium, zinc, and copper intake and status of vegetarian, vegan, and omnivore children and adolescents: results of the VeChi youth study

**DOI:** 10.1007/s00394-025-03803-w

**Published:** 2025-10-27

**Authors:** Rebecca Simon, Elisa Richter, Kristina Lossow, Morwenna Fischer, Alfred Längler, Andreas Michalsen, Stine Weder, Markus Keller, Anna P. Kipp, Ute Alexy

**Affiliations:** 1 Nutritional Physiology, Institute of Nutritional Sciences, Friedrich Schiller University, Jena, Germany; 2 Faculty of Human Resources, Health and Social Work, University of Applied Sciences (FHM), 33602 Bielefeld, Germany; 3 Department of Paediatrics, Gemeinschaftskrankenhaus, Herdecke, Germany; 4 Faculty of Health, Professorship for Integrativ Paediatrics, Witten Herdecke University, Witten, Germany; 5 Institute of Social Medicine, Epidemiology and Health Economics, Charité, Universitätsmedizin Berlin, 10117 Berlin, Germany; 6 Research Institute for Plant-Based Nutrition, 35444 Gießen/Biebertal, Germany; 7 Department of Nutritional Epidemiology, Institute of Nutritional and Food Science, University of Bonn, 44225 Dortmund, Germany

**Correction to**: **European Journal of Nutrition (2025) 64:247**. 10.1007/s00394-025-03761-3

In the original version of this article, table 2 was splitted into 2 parts (the second part without title spans) mistakenly. The correct table 2 should have appeaed shown below

**Table 2:** Adjusted means, standard errors (SE), and adjusted mean differences (%) of trace element (selenium, copper, zinc) biomarker and dietary intake data between vegetarian, vegan, and omnivore children and adolescents (6–18 years) from the Vechi youth study for the total sample (*n* = 324) and stratified by puberty (pre-pubertal *n* = 117/pubertal *n* = 205)

	Vegan	Vegetarian	Omnivore	Vegan^2^		Vegetarian^2^	
	Adjusted ^1^ mean (SE)	Adjusted ^1^ mean (SE)	Adjusted ^1^ mean (SE)	Δ (95% CI)	p-value	Δ (95% CI)	p-value
**Biomarker**							
*Selenium* (µg/L)							
Total Sample	64.1 (1.5)	62.9 (1.7)	70.8 (1.5)	– 0.11 (– 0.17; – 0.06)	**0.0004**	– 0.15 (– 0.21; – 0.09)	**0.0004**
Pre-pubertal	58.1 (2.4)	59.3 (2.6)	69.0 (2.2)	– 0.17 (– 0.26; – 0.09)	**0.0004**	– 0.17 (– 0.26; – 0.08)	**0.001**
Pubertal	67.3 (2.0)	65.2 (2.4)	71.8 (2.0)	– 0.08 (– 0.15; – 0.02)	**0.0277**	– 0.14 (– 0.21; – 0.07)	**0.0007**
*SELENOP * (mg/L)							
Total Sample	2.70 (66)	2.31 (78)	3.24 (66)	– 0.20 (– 0.27; – 0.13)	**0.0004**	– 0.39 (– 0.46; – 0.30)	**0.0004**
Pre-pubertal	2.75 (118)	2.44 (131)	3.27 (113)	– 0.18 (– 0.30; – 0.07)	**0.0056**	– 0.34 (– 0.46; – 0.21)	**0.0004**
Pubertal	2.71 (85)	2.25 (100)	3.24 (82)	– 0.20 (– 0.29; – 0.11)	**0.0004**	– 0.42 (– 0.51; – 0.32)	**0.0004**
*GPX3 * (U/L)							
Total Sample	166 (3)	165 (4)	178 (3)	– 0.07 (– 0.13; – 0.01)	**0.0330**	– 0.09 (– 0.15; – 0.02)	**0.0185**
Pre-pubertal	163 (6)	169 (7)	172 (6)	– 0.06 (– 0.16; 0.04)	0.3015	– 0.01 (– 0.12; 0.09)	0.1153
Pubertal	171 (4)	162 (5)	182 (4)	– 0.07 (– 0.15; 0.01)	0.1146	– 0.13 (– 0.21; – 0.04)	**0.0074**
*Copper* (µg/L)							
Total Sample	942 (26)	950 (31)	943 (26)	– 0.00 (– 0.06; 0.06)	0.9340	0.00 (– 0.07; 0.07)	0.9362
Pre-pubertal	936 (37)	1026 (42)	1043 (36)	– 0.11 (– 0.20; – 0.01)	**0.0500**	0.01 (– 0.09; 0.11)	0.9340
Pubertal	927 (35)	874 (42)	884 (34)	0.03 (– 0.05; 0.11)	0.5782	-0.02 (– 0.10; 0.07)	0.8074
CPO (U/L)							
Total Sample	110 (4)	107 (5)	109 (4)	0.02 (– 0.07; 0.10)	0.8074	– 0.02 (– 0.11; 0.08)	0.8074
Pre-pubertal	112 (5)	119 (6)	125 (5)	– 0.13 (– 0.26; 0.01)	0.1021	– 0.02 (– 0.17; 0.12)	0.8074
Pubertal	106 (5)	96 (6)	100 (5)	0.07 (– 0.05; 0.18)	0.3234	– 0.03 (– 0.15; 0.10)	0.8054

Zinc (µg/L)							
Total Sample	822 (15)	795 (18)	873 (15)	– 0.06 (– 0.10; – 0.01)	**0.0270**	– 0.08 (– 0.12; -0.03)	**0.0048**
Pre-pubertal	797 (25)	776 (28)	849 (24)	– 0.07 (– 0.14; 0.01)	0.1139	– 0.08 (– 0.16; 0.01)	0.1139
Pubertal	836 (20)	811 (23)	885 (19)	– 0.05 (– 0.10; 0.01)	0.1172	– 0.08 (– 0.13; – 0.02)	**0.0270**
**Intake**							
Selenium (µg/d)	31.0 (2.7)	38.1 (3.2)	44.6 (2.7)	– 0.25 (– 0.34; – 0.16)	**0.0004**	– 0.07 (– 0.17; 0.03)	0.2593
Copper (mg/d)	1.6 (0.2)	2.2 (0.2)	1.7 (0.2)	0.17 (0.10; 0.23)	**0.0004**	0.49 (0.41; 0.56)	**0.0004**
Zinc (mg/d)	8.1 (0.3)	9.2 (0.3)	9.1 (0.3)	– 0.06 (– 0.11; – 0.00)	0.0852	0.07 (0.00; 0.13)	0.0646

^1^ Means (SE) adjusted for age and sex, GPX3 glutathione peroxidase-3 activity, SELENOP selenoprotein P, CPO ceruloplasmin oxidase activity.

^2^ Outlier of outcome variables were winsorized. Outcome variables have been log-transformed to account for skewness of raw data and to fulfill model requirements. Multiple imputation was used to account for missing covariables data, model adjusted for age, sex, puberty (pre-pubertal/pubertal) and socioeconomic status, models for intake were also adjusted for total energy intake per day. *p*-values were adjusted for multiple testing using the False Discovery Rate method (Proc Multitest in SAS).

The original article has been corrected.

