# Supplementary material for: Selenium, zinc, and copper intake and status of vegetarian, vegan, and omnivore children and adolescents: results of the VeChi youth study
Source: Eur J Nutr. 2025 Jul 22;64(5):247. doi: 10.1007/s00394-025-03761-3 (PMC12283858; doi:10.1007/s00394-025-03761-3)
Supplement: Supplementary file 1 — Supplementary Material 1 [file 394_2025_3761_MOESM1_ESM.docx]

Supplemental Material

Table S1: Unadjusted biomarker level and dietary intake of trace elements

Supplementary Table 1. Unadjusted median (Q1; Q3) of dietary intake and biomarkers of trace elements (selenium, copper, zinc) collected 2017-2019 in a sample of vegetarian, vegan, and omnivore children and adolescents (n= 324, 6-18 years) from the VeChi Youth Study.

|  | Vegan | Vegetarian | Omnivore |
| --- | --- | --- | --- |
| **Biomarker** | | | |
| Selenium (µg/L) | 55.8 (51.1; 69.2) | 61.3 (53.7; 70.5) | 68.2 (61.3; 77.5) |
| GPX3 (U/L) | 168 (133; 194) | 166 (137; 191) | 176 (155; 199) |
| SELENOP (µg/L) | 2116 (1748; 2625) | 2582 (2209; 3163) | 3185 (2765; 3674) |
| Copper (µg/L) | 884 (763; 1043) | 907 (763; 1034) | 896 (772; 1056) |
| CPO (U/L) | 98 (84; 122) | 101 (87; 124) | 105 (82; 131) |
| Zinc (µg/L) | 776 (706; 861) | 803 (714; 894) | 845 (770; 928) |
| **Intake** | | | |
| Selenium (µg/day) | 31.6 (24.7; 41.0) | 27.0 (19.6; 36.4) | 38.7 (26.3; 52.0) |
| Copper (mg/day) | 2.0 (1.7; 2.5) | 1.5 (1.2; 1.8) | 1.2 (1.0; 1.7) |
| Zinc (mg/day) | 8.1 (6.7; 10.2) | 7.8 (6.0; 9.4) | 8.5 (6.6; 11.4) |

GPX3 glutathione peroxidase-3 activity, SELENOP selenoprotein P, CPO ceruloplasmin oxidase activity
